# Natural food intake patterns have little synchronizing effect on peripheral circadian clocks

**DOI:** 10.1186/s12915-020-00872-7

**Published:** 2020-11-06

**Authors:** Xiaobin Xie, Ayaka Kukino, Haley E. Calcagno, Alec M. Berman, Joseph P. Garner, Matthew P. Butler

**Affiliations:** 1 Oregon Institute of Occupational Health Sciences, Oregon Health & Science University, 3181 SW Sam Jackson Park Road – L606, Portland, OR 97239 USA; 2 Current Address: Key Laboratory of Protein Modification and Degradation, School of Basic Medical Sciences, Guangzhou Medical University, Guangzhou, 511436 China; 3 Department of Comparative Medicine, Stanford University, Stanford, CA USA; 4 Department of Behavioral Neuroscience, Oregon Health & Science University, Portland, OR USA

**Keywords:** Meal timing, Peripheral clocks, Liver, Kidney, TRF, Time-restricted feeding, Period circadian proteins, Activity cycles, Constant darkness

## Abstract

**Background:**

Circadian rhythms across mammalian tissues are coordinated by a master clock in the suprachiasmatic nucleus (SCN) that is principally entrained by light-dark cycles. Prior investigations have shown, however, that time-restricted feeding (TRF)—daily alternation of fasting and food availability—synchronizes peripheral clocks independent of the light-dark cycle and of the SCN. This has led to the idea that downstream peripheral clocks are entrained indirectly by food intake rhythms. However, TRF is not a normal eating pattern, and it imposes non-physiologic long fasts that rodents do not typically experience. Therefore, we tested whether normal feeding patterns can phase-shift or entrain peripheral tissues by measuring circadian rhythms of the liver, kidney, and submandibular gland in *mPer2*^*Luc*^ mice under different food schedules.

**Results:**

We employed home cage feeders to first measure ad libitum food intake and then to dispense 20-mg pellets on a schedule mimicking that pattern. In both conditions, PER2::LUC bioluminescence peaked during the night as expected. Surprisingly, shifting the scheduled feeding by 12 h advanced peripheral clocks by only 0–3 h, much less than predicted from TRF protocols. To isolate the effects of feeding from the light-dark cycle, clock phase was then measured in mice acclimated to scheduled feeding over the course of 3 months in constant darkness. In these conditions, peripheral clock phases were better predicted by the rest-activity cycle than by the food schedule, contrary to expectation based on TRF studies. At the end of both experiments, mice were exposed to a modified TRF with food provided in eight equally sized meals over 12 h. In the light-dark cycle, this advanced the phase of the liver and kidney, though less so than in TRF with ad libitum access; in darkness, this entrained the liver and kidney but had little effect on the submandibular gland or the rest-activity cycle.

**Conclusions:**

These data suggest that natural feeding patterns can only weakly affect circadian clocks. Instead, in normally feeding mice, the central pacemaker in the brain may set the phase of peripheral organs via pathways that are independent of feeding behavior.

## Background

Healthy physiology is characterized by ~ 24 h circadian rhythms in all tissues that are entrained (synchronized) to cyclic environmental cues. Disruptions of the circadian system, such as by jet lag and shift work, increase the risk of poor health including obesity, diabetes, and heart disease in both humans and animal models [1–7]. Circadian disruptions cause adverse health outcomes via internal misalignment (in which internal clocks become desynchronized from each other) and external misalignment (in which clocks become desynchronized from external entraining cues) [6, 8–14].

Internal clock alignment is normally maintained by the central pacemaker in the suprachiasmatic nucleus (SCN) of the hypothalamus [15, 16]. The SCN is entrained by light, and in turn, it coordinates the timing of peripheral clocks via several potential pathways, one of which is thought to be the SCN’s control of eating behavior. In studies of time-restricted feeding (TRF), during which rodents are typically exposed to 4–12 h of food availability and 12–20 h of fasting each day, the feeding schedule reliably shifts the circadian phase of peripheral organ clocks, while the SCN remains entrained to the light-dark cycle [17, 18].^1^ This has led to a general model of circadian entrainment in which the SCN controls the phase of peripheral clocks via its control of feeding behavior.

**Fig. 1:**
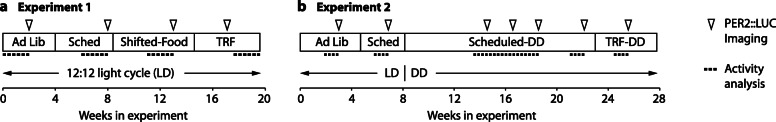
Timeline for both experiments, showing the time of baseline intake measures, food provision schedules (Ad Lib, Scheduled, Shifted, or TRF), the light-dark cycle (LD or DD), and points at which PER2::LUC imaging was conducted to measure the phase of peripheral organ circadian rhythms.

TRF imposes long fasting intervals that do not typically occur when food is available ad libitum, and it is not known whether natural eating patterns play a role in entraining peripheral clocks. To test this, we measured peripheral organ rhythms in simulated natural feeding conditions in light-dark cycles and in constant dim light (Fig. 1, Additional File 1: Fig. S1). We focused on the phase of the liver and kidney, two tissues that are entrained by TRF, and the submandibular gland, a tissue with a high amplitude oscillator that is insensitive to TRF and instead entrains to the light-dark cycle [17–20]. Surprisingly, we found that simulated natural feeding had little effect on peripheral clocks: these oscillators generally remained entrained to either the light-dark cycle or to the animal’s own rest-activity cycle.

## Results

### Experiment 1: Scheduled feeding in light-dark cycles

**Fig. 2:**
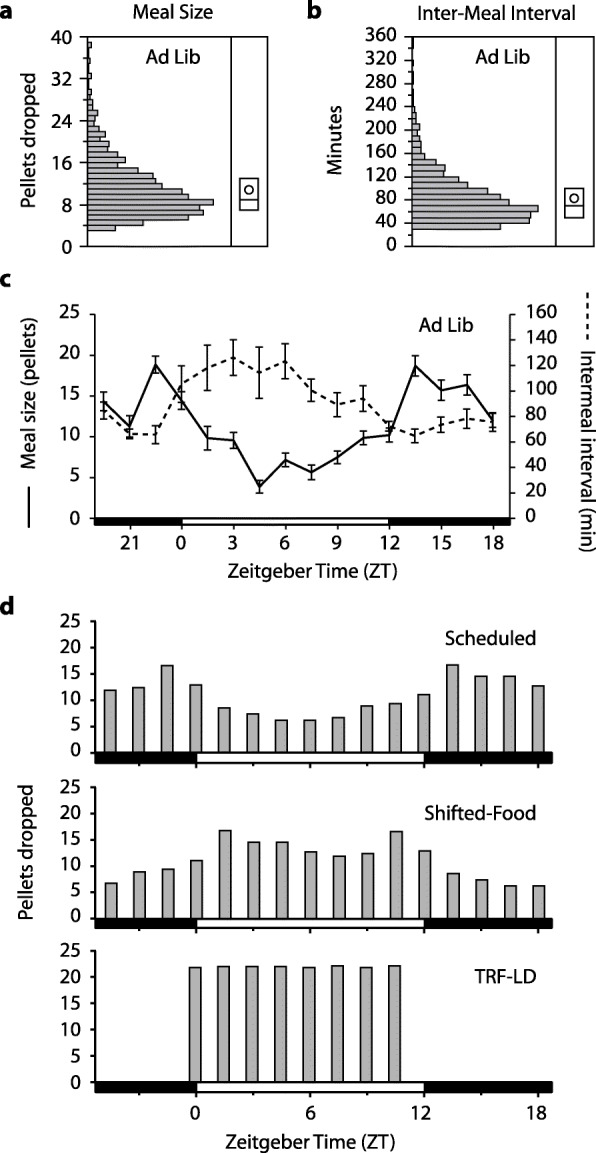
**a**, **b** Distributions of Ad Lib meal sizes in 20-mg pellets and inter-meal intervals in minutes (*n* = 10). The box plot shows the median and the 25th and 75th percentile; the mean is shown by the circle. **c** Both meal size and inter-meal interval varied with the time of day during Ad Lib conditions. The light-dark cycle is shown at the bottom, with Zeitgeber Time 0 (ZT0) and ZT12 defined as lights-on and lights-off, respectively. **d** The mean pellet drop schedules are plotted for Scheduled, Shifted-Food (12 h), and TRF conditions, all conducted in LD

We first tested whether simulated natural food intake patterns were sufficient to shift locomotor activity or peripheral clocks in a light-dark cycle (LD). After acclimating male mice to custom in-cage automatic feeders (Additional File 1**:** Fig. S2), the average Ad-Lib intake pattern was calculated. Both meal size and inter-meal interval varied across the light-dark cycle (Fig. 2a–c), with 65% of food intake during the dark phase (nocturnality index = 1.9). The mean meal size was 10.8 pellets (216 mg) (SD 6.2 pellets), and the mean inter-meal interval was 83 min (SD 62). To simulate natural intake patterns, inter-meal intervals were set to 90 min, and the amount provided in each meal varied (Fig. 2d, Additional File 1: Fig. S3). Note that because meals were defined by at least 3 pellets, the inter-meal intervals here are longer than the fasting intervals (see the Discussion section). The group average profile was applied to all mice, but the amounts were adjusted to each mouse’s baseline intake. To ensure that mice ate food when it was provided, the total food during Scheduled conditions was reduced by 5% compared to Ad Lib conditions. Body weight was maintained at 99–103% of baseline thereafter (Additional File 1: Fig. S4). The scheduled food was later shifted 12 h (Shifted-Food-LD). Finally, mice were switched to time-restricted feeding (TRF-LD), with eight equally sized meals spread across the light portion of the photocycle (Fig. 2d).

**Fig. 3:**
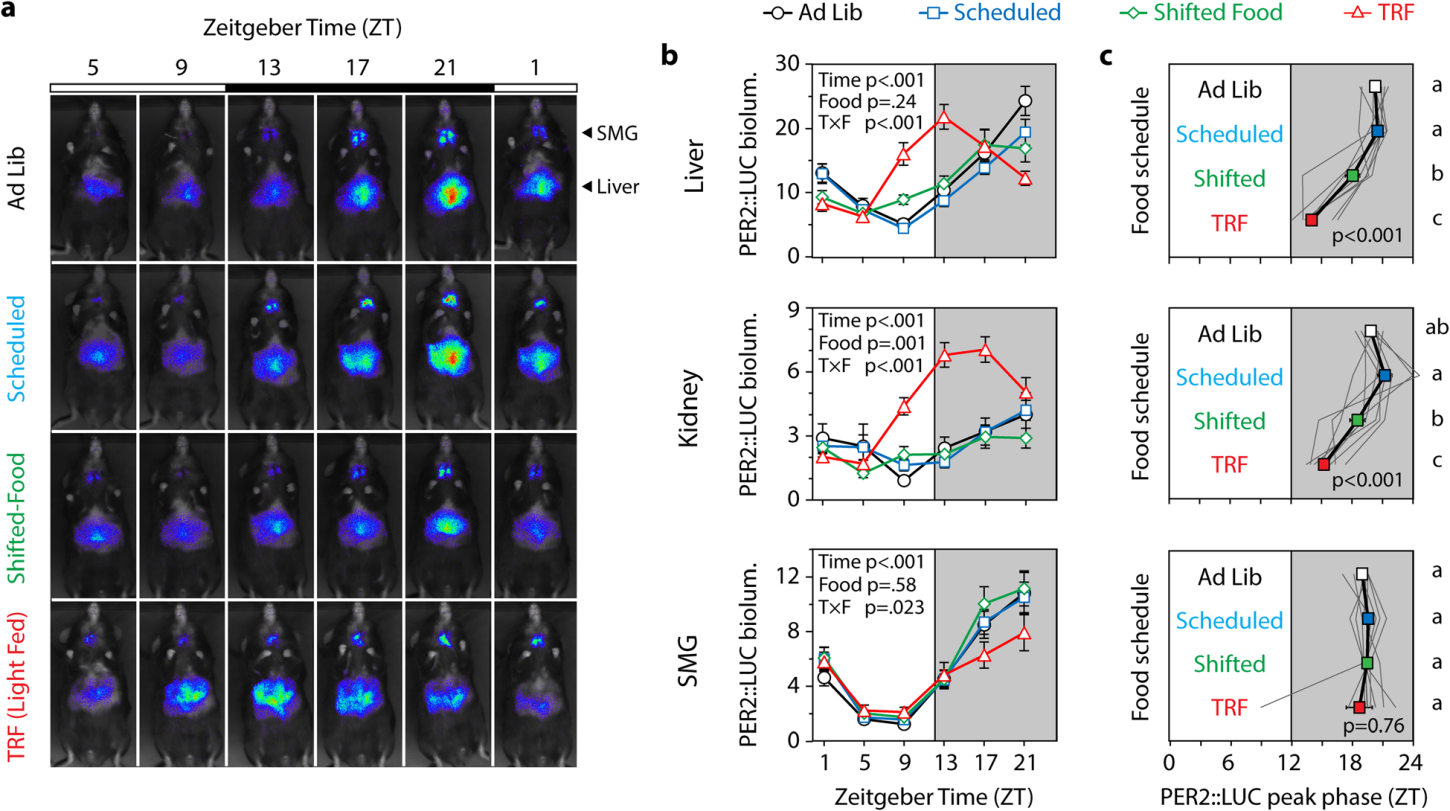
In vivo imaging and peripheral tissue phases. Every 4 h, mice were imaged after being lightly anesthetized and injected s.c. with luciferin. **a** Ventral images from a representative mouse: note that peak PER2::LUC bioluminescence in the SMG occurs at ZT21 in all conditions, whereas peak bioluminescence in the liver occurs earlier only in the TRF light-fed condition. **b** Mean bioluminescence is plotted as a function of time of day (*n* = 9). Statistical results from a 2-way repeated measures ANOVA are shown (main and interaction effects of zeitgeber time and food condition). A significant interaction suggests a change in the phase or amplitude of the rhythm. **c** The phase of each tissue was calculated from the direction of the mean resultant vector and plotted (± SE). *p* value indicates the result of a 1-way repeated measures ANOVA (feeding condition as the independent variable). Conditions that do not share a letter are significantly different (Tukey test, *p* < .05). Individuals are shown by gray traces

PER2::LUC bioluminescence rhythms from the liver, kidney, and submandibular gland were assessed in vivo at baseline and during each different feeding schedule (Fig. 3; raw data in Additional File 3). In the group-level analysis, bioluminescence peaked during the night in all tissues in Ad Lib, Scheduled, and Shifted-Food conditions, suggesting that shifting the food schedule had little phase-resetting effect on these peripheral clocks.

To capture individual variability, peak phase was calculated for each mouse and tissue (Additional File 1: Figs. S5, S6). In this analysis, there was a significant effect of feeding condition on liver phase (repeated measures ANOVA, *F*_3,24_ = 44.5, *p* < .001) and kidney phase (*F*_3,24_ = 19.6, *p* < .001), but there was no effect on the submandibular gland (*F*_3,24_ = 0.39, *p* = .76) (Fig. 3c). Pairwise comparisons showed that Scheduled meals did not change clock phase relative to Ad Lib conditions (Tukey test, n.s.). When the Scheduled feeding was shifted 12 h (Shifted-Food), peripheral tissues continued to peak during the dark, with only small shifts in the liver (2.5 ± 0.7 h) and kidney (2.8 ± 1.1 h) (Tukey test, *p* < .05) and no advance in the submandibular gland (Fig. 3c, 0.1 ± 0.4 h, n.s.). The kidneys of one mouse and the liver of another shifted robustly to the new food schedule. Without these, the group phase shifts were less than 2 h. Conclusions based on circular statistics were similar, with the exception that the Shifted-Food schedule did not significantly advance the kidney clock (Additional File 1: Fig. S5, Mardia-Watson-Wheeler test, *p* > .30). These data suggest that naturalistic food intake patterns are not strong enough zeitgebers (time-setting cues) to phase-shift the peripheral circadian clock system.

The lack of food entrainment was surprising given previous reports from TRF-treated mice [17, 18, 20–22], so mice were switched to a modified TRF cycle, with 8 equally sized meals presented during the light phase (ZT0–12). After 2 weeks, this schedule was sufficient to significantly advance the liver and kidney clocks relative to the first Scheduled condition by 6.5 and 6.0 h (Fig. 3c, Additional File 1: Fig. S5). TRF had no effect on submandibular gland phase.

During different food conditions, mice remained nocturnal as expected [21, 23]. The nocturnality index (nocturnal to diurnal activity ratio) was significantly greater than 1 in all conditions, though there was a significant reduction in this index during the last TRF-LD condition (Additional File 1: Figs. S7, S8).

### Experiment 2: Scheduled feeding in constant darkness

In experiment 1, shifting the simulated natural food intake schedule did not appreciably shift locomotor activity or peripheral tissue clock phase. But the zeitgeber strength necessary to shift clocks may be much higher than to maintain entrainment, for example, as observed in responses to light [24]. Therefore, experiment 2 was conducted on a new cohort of mice to determine if simulated natural intake patterns were sufficient to entrain the liver clock in the absence of a light-dark cycle.

**Fig. 4:**
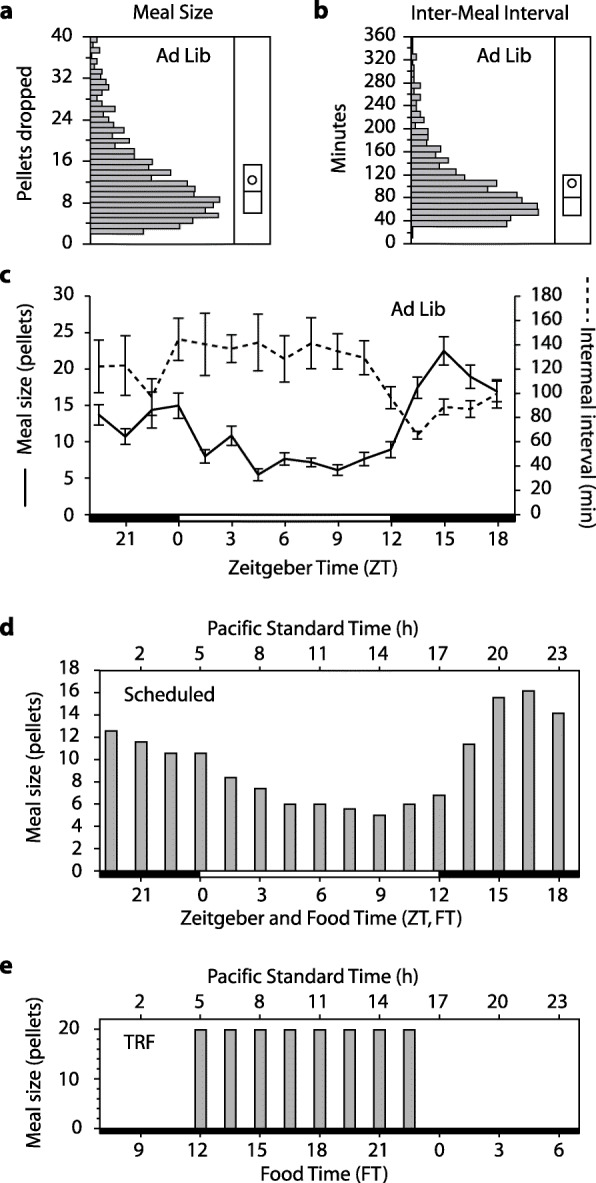
**a**, **b** Experiment 2 meal size and inter-meal interval distributions as in Fig. 2. **c** Both meal size and inter-meal interval varied with time of day during ad lib conditions. **d** The mean pellet drop schedule based on *n* = 12 mice is shown; the peak in intake at the end of the night was not as pronounced as in experiment 1. Both ZT12 and FT12 occur at 1700 PST. **e** After free-running in darkness with scheduled feeding, mice were switched to TRF conditions with 12-h food availability (FT12 at 0500 PST)

Mice exhibited a normal feeding pattern with 68% of food consumed during the dark (nocturnality index = 2.1, Fig. 4). The mean intake profile for the second cohort of mice was again applied using a 90-min inter-meal interval. Body weight stayed at 99–109% of baseline during the experiment (Fig. S9), and transitioning from Ad Lib to Scheduled feeding in LD did not phase shift the circadian system (Fig. S10).

**Fig. 5:**
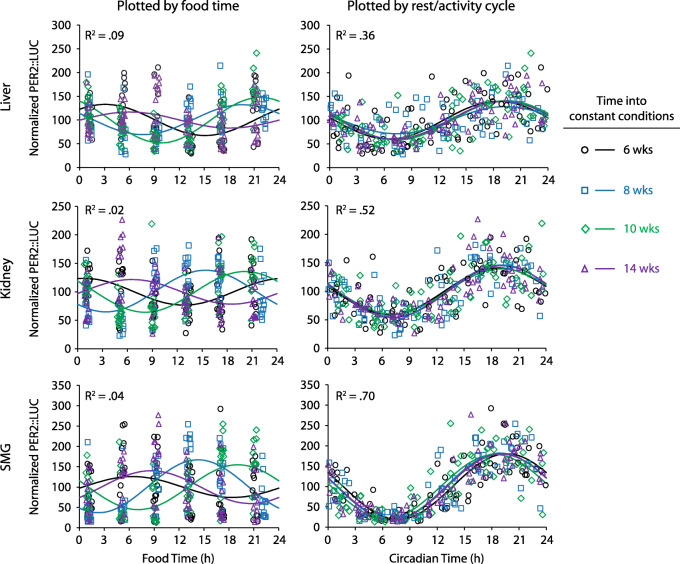
PER2::LUC bioluminescence was measured in vivo 6 times/day (*n* = 12 mice). This was done after 6 (circle), 8 (box), 10 (diamond), and 14 (triangle) weeks of housing in constant conditions. The same data are plotted against either the timing of food (left) or the timing of each animal’s rest-activity cycle (right). Curves are the best fit cosines for the 4 measurement days. *R*^2^ values are from the full model that combined all four measurement days (Additional File 2: Table S1). The three different tissues are all much better aligned with circadian rhythms of rest/activity, and thus with the brain’s pacemaker, than with the schedule of food intake

Without changing the Scheduled food condition, the mice were released into constant dim red light (DD, 0.1 lux), and their activity rhythms began to free-run according to their endogenous non-24-h period. This immediately established that simulated natural food patterns do not entrain locomotor activity rhythms. In DD, activity phase and peripheral organ phase were calculated periodically over 3 months, and the PER2::LUC bioluminescence was analyzed as a function of the rest-activity cycle (circadian time, with activity onset defined as CT12) or food time (FT12–24 representing the period of greatest intake/provision and corresponding to the original 1700–0500 dark period) (Fig. 5). PER2::LUC expression was synchronized to the rest-activity cycle: circadian time explained 36–70% of the variance in the model (Additional File 2: Table S1). In contrast, peripheral rhythms were desynchronized when plotted against the food intake cycle, and food time only explained 2–9% of the variance.

Over the four tests of phase during Scheduled-DD conditions, each tissue’s phase was calculated and plotted circularly as a function of food time or circadian time (Additional File 1: Fig. S11). Scheduled food did not entrain the peripheral organs: from test to test, the phase in food time varied widely (Mardia-Watson-Wheeler test, *W* ≥ 14.9, *p* ≤ .021). In contrast, peripheral tissues were always in phase with circadian time with little test-to-test variability (*W* ≤ 10.7, *p* ≥ .099).

**Fig. 6:**
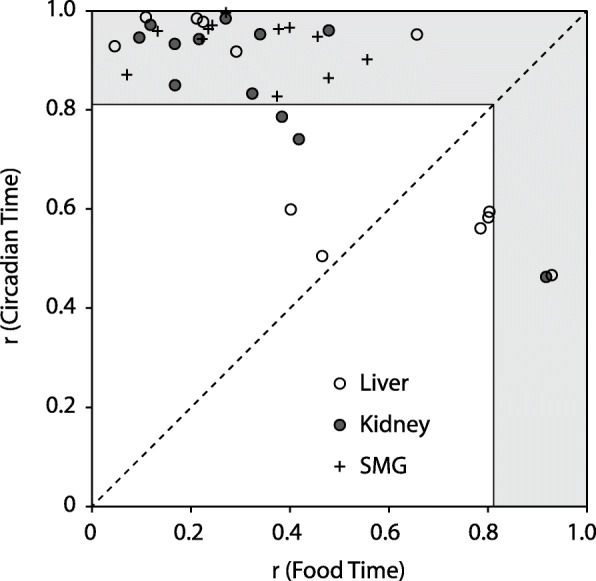
Individual variability in liver and kidney entrainment to food. For each tissue in each mouse, the preferred phase and *r* value were calculated from the four phase measures completed in constant conditions. This was done both in food time and in circadian time. The two *r* statistics are plotted against each other. Points above the diagonal dotted line indicate that circadian time better predicts phase than food time. Points that fall in the gray-shaded area have a significant preferred phase (*r* > 0.82; *p* < 0.05; Rayleigh test for *n* = 4). Note: the liver and kidney with significant phase clustering by food time are not from the same mouse. Activity and tissue phases for the mouse with significant liver food time (empty circle on right) are shown in Additional File 1: Fig. S12A

There was evidence for individual variability in sensitivity to food cues (Additional File 1: Fig. S12). For each tissue, we tested whether circadian phase clustered better along food time or circadian time by calculating the overall mean resultant vector from the four phases measured during constant conditions (4 repeated measures of phase per tissue). The submandibular glands did not entrain to food and always showed significant clustering in circadian time (Fig. 6, *r* ≥ .83, *p* < .05 for all). In contrast, phase in some kidneys and livers was sensitive to food intake patterns. One kidney and one liver (not from the same mouse) showed significant phase clustering in food time, indicating food entrainment. Nevertheless, 8/12 livers and 11/12 kidneys showed stronger clustering by circadian time (above the dotted line in Fig. 6); this was significant in 6/12 livers and 9/12 kidneys.

After 15 weeks in Scheduled-DD conditions, mice were switched to TRF with 12 h of feeding and 12 h of fasting, to determine whether restricted feeding with a long daily fast could entrain rhythms in DD. As in experiment 1, mice were provided 8 equally sized meals presented during the time of the original photophase (0500–1700 PST). Period shortened in the majority of mice, but locomotor activity entrained (period = 24 h) in only two mice (Additional File 1: Fig. S13).

Though food schedules did not entrain locomotor activity in most mice, TRF did entrain rhythms of the liver and kidney (Additional File 1: Fig. S14). When tissue phases were plotted by food time, the liver and kidney phases strongly clustered around FT0 (Rayleigh test, *p* < .001), similar to phases observed during Ad Lib conditions. Though the SMG displayed statistically significant clustering, there was a wide range of phases. The opposite results were obtained when plotted by circadian time: strong clustering for the SMG and weak clustering for the liver and kidney. Therefore, TRF in DD effectively entrained kidney and liver but did not entrain either the SMG or locomotor activity.

## Discussion

We show here that natural feeding behavior has little resetting effect on peripheral clocks. In experiment 1, a 12-h shift of the simulated natural food provision schedule resulted in small shifts in the liver and kidney and no shift in the SMG. In experiment 2, we removed the light-dark cycle and tested whether the scheduled food would continue to entrain peripheral clocks. Peripheral organ phase was nevertheless much better predicted by the rest-activity cycle; food significantly entrained only 1 of 12 kidneys and 1 of 12 livers. As a positive control, TRF was an effective zeitgeber in both experiments, unlike the simulated natural feeding. From these data, we infer that under normal conditions of food intake, the SCN remains the dominant synchronizer of peripheral clocks and that this does not require the intermediate step of feeding behavior.

In TRF experiments, food phase-shifts peripheral organs independent of the light-dark cycle: when mice have free access within the food availability windows, food schedules that are 12 h apart cause 12-h differences in peripheral clock phase [17, 18]. This has led to a model of peripheral entrainment in which the SCN entrains patterns of behavior (activity and food intake), and these behaviors and their metabolic sequelae in turn entrain peripheral organs. In this experiment of simulated natural feeding, however, the effects of food intake rhythms on peripheral clock phase were small. This may be because natural intake patterns of rodents do not feature long fasting intervals. Even in experiments with varied meal size and frequency, peripheral organ phase generally tracks with the longest fast (ranging from 8 to 12 h; [25]). The extended fast may trigger phase resetting via the drop in circulating insulin [26]. The long fasts also appear important for the metabolic health benefits of TRF in rodents and humans [21, 27–30].

Fasting intervals were longer during the day than the night, so by setting a constant inter-meal interval (90 min), we may have removed a physiologically relevant fasting signal. The shortest fast needed to entrain peripheral clocks is not known, but is likely 4–8 h, given that 8-h but not 4-h fasts alter liver clock gene expression [26]. We therefore analyzed the distribution of fasting intervals during Ad Lib conditions to determine how often > 4 h fasts occur. True fasting intervals (no pellets dropped) are shorter than the inter-meal intervals, since mice occasionally consumed 1–2 pellets, and this was not counted as a meal. The maximum average fasting intervals per bin in the two experiments were 93 min (ZT4.5–ZT6) and 91 min (ZT3–4.5), similar to the 90-min inter-meal interval imposed on the mice during scheduled feeding and shorter than the peak mean inter-meal intervals (120–140 min, Figs. 2 and 4). Of course, the averages may not be informative if individual mice still have long fasts each day. The incidence of long fasts is shown in Additional File 2: Tables S2-S3. Over 80 mouse-days under Ad Lib conditions in experiment 1, only 13 intervals lasted longer than 4 h (0.7% of all fasting intervals). In experiment 2, there were 53 such fasts in 120 mouse-days (2.3%). Based on TRF experiments, it would be the long fasts during the light phase that would entrain normal night-time peaks of PER2::LUC in the peripheral organs. These were not equally distributed across mice. Though two mice in experiment 2 had almost one long fast per day, the other 20 mice had long fasts on half or fewer of their days and 5 never experienced a light-phase fast > 4 h. These data match other reports that voluntary inter-meal intervals in mice rarely exceed 5 h [31]. We therefore conclude that naturally occurring fasting intervals during Ad Lib feeding are unlikely to entrain peripheral clocks.

Our results extend conclusions from studies in which food rhythm effects are experimentally removed by providing rats and mice with equally spaced meals around the clock [25, 32–34]. These show that food rhythms are not necessary to maintain peripheral rhythm synchrony and that in the absence of food rhythms, adrenal hormone signaling may link central and peipheral clocks [33]. But, during normal feeding conditions, it is still not known whether food-independent cues are the primary mechanisms of entrainment or are complementary to food-derived cues as suggested by TRF experiments. In experiment 2, we simultaneously tested the competing effects of natural food intake cues versus rest-activity cycle-related cues, in a manner similar to forced desynchrony and T-cycle studies [14, 35]. Our finding that SCN cues dominate is novel; this extends conclusions based on experiments in which the food rhythm was eliminated. The SCN communicates time of day information via neural, endocrine, and physiological pathways [15, 16]. Potential entrainment mechanisms include glucocorticoids [33, 36–38], autonomic nervous system projections [39, 40], body temperature [41, 42], and melatonin [43, 44].

Two results from the TRF conditions used here bear discussion: peripheral phase in experiment 1 and the differential entraining effect on clocks in experiment 2. First, in experiment 1, the liver and kidney shifted ~ 6 h, substantially less than the ~ 12 h expected based on restricted feeding [17, 18, 45] and which we have observed when mice can eat ad libitum during food availability periods [20]. Two important differences (as yet untested) are (a) the use of set meal sizes that prevent the initial gorging response to food availability that is observed in light-fed but not dark-fed mice [23] and (b) the imposed short fasts during food availability. Similar shifts to what we observed (~ 6 h) were also reported for mice fed 3 equal-sized meals per day in the light phase [25]. Together, these data suggest that the full 12-h resetting observed in day-fed mice eating ad libitum requires both long fasts and a large refeeding response. Discrepant phase shifting in different types of TRF protocols emphasizes that the effects of food on the circadian system can vary from quite weak—as observed in this work—to very strong depending on the meal time, the duration of fast, and the pattern of eating when food is available. Second, in experiment 2, locomotor activity rhythms continued to free-run in a majority of mice despite the 12 h feeding schedule during TRF-DD. This is consistent with some reports (providing 2–6 h of food availability per day) [35, 46, 47] and differs from others (4–8 h of food/day) [48, 49]. Others have shown an intermediate proportion of mice that entrain to food (44–67%) [50, 51]. Both strain differences and duration of daily food availability can contribute to this inconsistency [52]. The misalignment between liver/kidney (entrained to food) and activity/submandibular gland (free-running and synchronized to each other) further underscores that TRF has little effect on the central clock.

To what extent do our results shed light on human peripheral clock control? As yet, there are few investigations of this, because of the difficulty in measuring peripheral clock phase in humans [53]. Studies of circulating factors have established that a shift in meal time can shift the phase of glucose and other metabolite rhythms, but the associated phase shifts in clock gene expression in white blood cells are much smaller [54, 55]. This suggests that meals are weak zeitgebers for human clocks even as they drive daily rhythms in metabolic physiology. Even though these studies included daily fasts of 12–14 h, the weak response of human clocks to shifting meal schedules resembles the response of our mice in experiment 1 and differs from the robust response seen in mice under TRF. To understand this, we must distinguish between meal intake (where TRF in mice can match human eating) and the metabolic effects that those meals elicit. Unlike in humans, an overnight fast in a mouse can reduce body weight by 15%, induce a catabolic state, and engage starvation protection mechanisms [31, 56]. Therefore, even though ad lib-fed mice eat at different rates and times, they may better model the gentler daily waxing and waning of circulating metabolic markers in humans.

This experiment was enabled by home cage automated feeders that allowed precise measurement of food intake and provision of a set pattern. The use of *mPer2*^*Luc*^ mice and in vivo imaging let us repeatedly sample circadian phase in multiple tissues in individuals, revealing some inter-individual variability in zeitgeber sensitivity. Glucose, insulin, and other potential resetting cues were not measured in this study, so although simulated natural food patterns did not alter behavior and peripheral organ rhythms compared to ad lib intake, whether and how the food schedule may have altered metabolic signaling remains unknown. Finally, the scheduled food pattern was imposed by varying meal sizes, so the contribution of inter-meal interval variability remains unknown.

## Conclusions

These two experiments show that natural food intake patterns have only a weak effect on the phase of peripheral clocks. This challenges a circadian model in which peripheral clocks are coordinated by the SCN via its control of food intake. Instead, our results suggest that during normal feeding conditions, the SCN entrains peripheral clocks via feeding-independent pathways.

## Methods

### Animals and housing

*mPer2*^*Luc*^ mice on a C57Bl/6 background, bearing a knockin fusion protein combining the clock gene *Period2* and a firefly luciferase reporter, were purchased from the Jackson Laboratories (B6.129S6-*Per2*^*tm1Jt*^/J, Strain Code: 006852) [57]. Male mice for this experiment were bred locally and maintained in a Thoren ventilated caging system in a pathogen-free barrier facility on pelleted cellulose bedding (BioFresh Performance Bedding, ¼” pelleted cellulose, Absorption Corp, Ferndale, WA), with food (LabDiet 5L0D) and water available ad libitum*.* For experiments, adult mice were transferred to an animal enclosure outside of the barrier facility, and housed individually in cages with a custom pellet feeder (Telos Discovery Systems, West Lafayette, IN, Additional File 1: Fig. S2), in a single cabinet with light-dark cycle control (Phenome Technologies, Lincolnshire, IL). The home cage feeder stands at one end of the cage (Thoren model #1, 19.6 cm × 30.9 cm × 13.3 cm), and takes up 7.5 cm, therefore limiting the living space length to 23.4 cm. Flat wire lids were used above the cage, and to prevent the water bottle from interfering with activity recording (see below), the water bottle was mounted at the end of the cage, with the sipper tube extending through the watering grommet (7.5 cm from the cage floor). Light was provided by green LEDs (525 nm, full width at half maximum (FWHM) 25 nm, 125 lux) from 0500 to 1700 PST. The use of narrowband monochromatic light simplifies studies of photoreceptor contribution to visual and non-visual responses [24]. To aid in husbandry and to ameliorate potential dim-light induced effects on the circadian system during imaging procedures, dim red light was on continually in the cabinet (625 nm, FWHM 25 nm, 0.2 lux). All procedures were approved by the Institutional Animal Care and Use Committee of Oregon Health & Science University.

### Feeding

At specified times, the feeders automatically dispensed 20 mg food pellets (Rodent Grain-Based Diet Sterile Product #S0163, Bio-Serv, Flemington, NJ). The presence of the pellet and head entries into the pellet trough (1.5 cm × 1.5 cm) were recorded by infrared beam breaks. During ad libitum feeding, a new pellet was dropped each time the pellet in the trough was removed. A failsafe mechanism drops another pellet 10 s later if the first pellet is not detected in the trough. Each dispensing event, head entry, and pellet removal was time-stamped and logged by an Access 2010 database (Microsoft, Seattle, WA); analyses were then conducted on binned counts (10 min bins). Individual meals were identified in the record by feeding bouts of at least three pellets (≥ 60 mg) with at least 20 min of fasting preceding and following the meal. Feeders were checked periodically to ensure that all pellets were eaten prior to the next meal and that no animals were hoarding food. Troughs were empty in 92% of 278 observations suggesting that the dispensing schedule determined the intake schedule.

### Locomotor activity

Activity counts were measured by a passive infrared detector (Phidgets 1111_0 motion sensor) mounted 16.5 cm above the cage; data were collected as counts per 10 min bin. Activity was used to calculate the nocturnality index (dark phase activity divided by light phase activity over 14 days). Additionally, the circadian period and phase were calculated from consecutive activity onsets over 7–10 days in ClockLab (Actimetrics, Wilmette, IL). Activity onset was defined as Circadian Time 12 (CT12). Period and phase during experiment 2 are included in Additional File 3.

### Protocol schedules

*Experiment 1*. The timeline of events is shown in Fig. 1a. At week 0, mice (21–23 weeks of age, *n* = 10) were transferred to the automatic feeding cages. Mice were maintained in ad libitum (Ad Lib) feeding conditions with a 12:12 light:dark (LD) schedule for 4 weeks. From weeks 4–9, mice were exposed to Scheduled feeding (Scheduled-LD) that simulated the group’s average feeding profile. At week 9, the scheduled feeding pattern was shifted by 12 h (Shifted-Food-LD). Finally, at week 15, mice were exposed to time-restricted feeding (TRF-LD) for 5 weeks, in which food was provided only during lights-on. Peripheral PER2::LUC bioluminescence rhythms were measured once in each feeding condition. For one mouse, a feeder failure introduced long fasts during the Scheduled and Shifted-Food conditions. The mouse recovered, but his data were excluded a priori from peripheral clock phase analyses, leaving an analytic dataset of *n* = 9. *Experiment 2*. Mice, aged 16–17 weeks (*n* = 12), were transferred to automatic feeding cages as above and fed Ad Lib for 5 weeks (Fig. 1b). Scheduled feeding was imposed at week 5, simulating the ad lib intake pattern for this new cohort (Scheduled-LD). From week 8, the mice were housed in constant dim red light (Scheduled-DD, 0.1 lux). Finally, they were shifted to TRF in DD (TRF-DD); food was available during the initial time of lights-on (0500–1700 PST). As above, peripheral organ rhythms were measured from PER2::LUC bioluminescence.

### Imaging protocol

An in vivo imaging system (Stanford Photonics, Stanford, CA) was used to measure PER2::LUC bioluminescence in the liver, submandibular gland, and kidney [20, 22, 25]; this system includes computer-assisted capture of bioluminescent images via an Electron Magnified (EM) CCD camera (ImageEM, Hamamatsu, Japan, controlled by Piper software version 2.6.89.18, Stanford Photonics) connected to an ONYX dark box (Stanford Photonics) in which mice can be maintained under isoflurane anesthesia on a 37 °C temperature-controlled stage (mTCII micro-Temperature Controller, Cell MicroControls, Norfolk, VA). Clock phase was determined from PER2::LUC bioluminescence measured every 4 h (6 measures in one day). Mice were anesthetized with 2–4% isoflurane to prevent movement during imaging. Prior to taking the first image, the fur was shaved from areas around the liver, submandibular gland, and kidneys. Mice were then injected s.c. (15 mg/kg) with d-luciferin potassium salt (Promega, Madison, WI) dissolved in sterile phosphate-buffered saline (30 mg/10 mL) and filtered (0.2 μm). The dorsal and ventral surfaces of the mice were imaged 8 and 10 min after luciferin injection, respectively. Bioluminescence was captured by the camera in EM mode (sum of eight 125-ms exposures, gain 500). A brightfield reference image was taken each time under dim red light (633 nm, FWHM 15 nm, 1.6 lux). After imaging, the animals were immediately returned to their cages for recovery. All bioluminescence data are included in Additional File 3.

Bioluminescence was scored offline. Each image was opened in ImageJ [58], and the intensity of the 24-bit grayscale image quantified using defined regions of interest, centered on the brightest areas of the tissue (liver, 6 mm × 6 mm; both kidneys, 25 mm × 20 mm; submandibular gland, 10 mm × 10 mm).

### Analyses and Statistics

Error is reported as SEM unless otherwise indicated, and all statistical tests were evaluated as two-sided.

#### Defining time

This experiment combined light cycles, food cycles, and free-running cycles, so data were analyzed as a function of zeitgeber time, food time, and circadian time, respectively (Additional File 1: Fig. S1). Zeitgeber time (ZT) is defined by the light-dark cycle with lights-off at ZT12 and lights-on at ZT0. Food time (FT) is defined similarly, with the major intake occurring from FT12 to FT0. For example, in Scheduled-LD conditions, FT12 = ZT12. During TRF, FT12 is defined as the start of food availability. Lastly, circadian time (CT) is specific to each animal’s rest/activity cycle, and CT12 is defined as the time of activity onset.

#### Assessing rhythms

The six in vivo bioluminescence measurements per 24 h were used to calculate a circadian profile and a peak phase. Because of variation in skin pigmentation and fur shaving, the six measures were normalized as per cent of the mean within mouse, tissue, and experimental day (Additional File 1: Fig. S6). *Circadian profile comparisons*: In experiment 1, circadian profiles of bioluminescence were analyzed by repeated measures ANOVA with food condition and zeitgeber time as independent factors. In experiment 2, bioluminescence patterns were analyzed by cosinor analysis as a function of circadian time or food time. A mouse’s activity rhythm free-runs in constant conditions, so circadian time drifted in and out of phase with the 24 h scheduled food time. The ability of circadian time versus food time to predict the peripheral organ phase was assessed by comparing the goodness of fit of the cosinor regression (*R*^2^ and AICc). *Phase analysis*: Circadian phase of individual organs was defined by the direction of the mean resultant vector (Additional File 1: Fig. S6); this provided point estimates of phase for each tissue in each mouse. Circular statistics were employed to determine whether there was a preferred phase in each condition (Rayleigh test) and whether the tissue phase differed between feeding conditions or between different weeks into DD (Mardia-Watson-Wheeler test) [59].

#### Individual differences

To analyze individual differences, phase clustering within mouse and within organ was assessed by the Rayleigh test (*n* = 4 phase determinations per mouse per organ in DD). The mean resultant vector lengths (*r*: *r* = 1 when all points occur at the same phase, *r* = 0 when points are uniformly distributed around the clock) were plotted to illustrate the strength of phase clustering when assessed by circadian time versus food time during DD (e.g., Fig. 6).

#### Other behaviors

Nocturnality was analyzed by ordinary regression on log-transformed activity with mouse as a repeated measure and is presented as median and 95% confidence interval. Groups are more nocturnal if the 95% confidence interval does not include 1. Changes in circadian period and phase were assessed by paired *t* test.

## Supplementary information

**Additional file 1: Fig. S1.** Schematic showing the different timing conventions: Pacific Standard Time, Zeitgeber Time, Food Time, Circadian Time. **Fig. S2.** Automatic feeder design. **Fig. S3.** Meal size and inter-meal interval variability. **Fig. S4.** Experiment 1 body weight. **Fig. S5.** PER2::LUC bioluminescence and circular statistics for phase in Experiment 1. **Fig. S6.** Example of phase determination from the mean resultant vector. **Fig. S7.** Representative behavior during Experiment 1. **Fig. S8.** Nocturnality index during Experiment 1. **Fig. S9.** Experiment 2 body weight. **Fig. S10.** Differences in phase between Ad Lib and Scheduled food conditions in Experiment 2. **Fig. S11.** Circular statistics for phase during constant conditions in Experiment 2. **Fig. S12.** Combined plots of peripheral clock phase and free-running locomotor activity. **Fig. S13.** Locomotor activity patterns in TRF in constant dim light. **Fig. S14.** Phase during TRF in Experiment 2.

**Additional file 2: Table S1.** Cosinor regression results for phase during scheduled feeding in constant dim light (Sched-DD) in Experiment 2. **Table S2.** Fasting intervals during Ad Lib feeding. **Table S3.** Number of long (> 4 h) fasts experienced by each mouse.

**Additional file 3.** PER2::LUC bioluminescence data. **Tab Data_Expt1**: PER2::LUC bioluminescence data in ZT and FT for Experiment 1. **Tab Dictionary_Expt1**: Data dictionary defining terms. **Tab Data_Expt2**: PER2::LUC bioluminescence data, locomotor activity period and phase, and image times in CT, FT, and ZT for Experiment 2. **Tab Dictionary_Expt2**: Data dictionary defining terms.

## Data Availability

The datasets generated and analyzed during the current study are available from the corresponding author on reasonable request.

## Abbreviations

AL: Ad libitum food access
CT: Circadian Time (see Additional File 1: Fig. S1)
DD: Constant dim red light
FT: Food Time (see Additional File 1: Fig. S1)
LD: Light-dark cycle
PER2::LUC: Period2::Luciferase fusion protein
PST: Pacific Standard Time
Sched: Scheduled food
SCN: Suprachiasmatic nucleus
SMG: Submandibular gland
TRF: Time-restricted feeding
ZT: Zeitgeber Time (see Additional File 1: Fig. S1)

## Footnotes

1 In TRF, food is usually provided ad libitum during the time it is available. In this work, we employed a modified TRF composed of a daily 12-h fast and food available in eight equal meals across 12 h.
