# Supplementary material for: Natural food intake patterns have little synchronizing effect on peripheral circadian clocks
Source: BMC Biol. 2020 Nov 6;18:160. doi: 10.1186/s12915-020-00872-7 (PMC7646075; doi:10.1186/s12915-020-00872-7)
Supplement: Supplementary file 2 — Additional file 2: Table S1. Cosinor regression results for phase during scheduled feeding in constant dim light (Sched-DD) in Experiment 2. Table S2. Fasting intervals during Ad Lib feeding. Table S3. Number of long (> 4 h) fasts experienced by each mouse. [file 12915_2020_872_MOESM2_ESM.pdf]

Additional File 2. Supplementary Tables

Natural food intake patterns have little synchronizing effect on peripheral circadian clocks

Xiaobin Xie, Ayaka Kukino, Haley E Calcagno, Alec M Berman, Joseph P Garner, Matthew P Butler

Table S1. In Experiment 2, normalized PER2::LUC bioluminescence across the four tests (weeks 6-14 in DD) was analyzed by cosinor regression with independent variables of circadian time (CT), food time (FT), or both (12 mice × 4 tests × 6 bioluminescence measures = 288 points per tissue). Mouse number was included as a random factor to account for repeated measures. The table shows goodness of fit and coefficient estimates for the different models. Regressions against CT outperformed regressions against FT as indicated by a larger R^2 and smaller AICc. Though the effect of adding FT to the model was small, it significantly improved the model fit (Partial F test based on reduced SSE, Liver, $F_{8,268}$ = 6.8, p<.001; Kidney, $F_{8,268}$ = 4.9, p<.001; SMG, $F_{8,268}$ = 3.7, p<.001). Including time into constant darkness as a fixed factor did not affect R^2 because mean bioluminescence was normalized to 100% within mouse and within experimental day.

	Fixed Effects Included in Model		
	CT	FT	CT+FT
Liver	R^2 = 0.36<br>AICc = 2869<br>cos(CT) $F_{1,285}$ = 12.1, p<.001<br>sin(CT) $F_{1,285}$ = 148, p<.001	R^2 = 0.09<br>AICc = 2969<br>cos(FT) $F_{1,285}$ = 28.0, p<.001<br>sin(FT) $F_{1,285}$ = 0.7, p=.40	R^2 = 0.47<br>AICc = 2810<br>cos(CT) $F_{1,283}$ = 19.0, p<.001<br>sin(CT) $F_{1,283}$ = 186, p<.001<br>cos(FT) $F_{1,283}$ = 56.5, p<.001<br>sin(FT) $F_{1,283}$ = 4.4, p=.037
Kidney	R^2 = 0.52<br>AICc = 2767<br>cos(CT) $F_{1,285}$ = 22.3, p<.001<br>sin(CT) $F_{1,285}$ = 284, p<.001	R^2 = 0.02<br>AICc = 2970<br>cos(FT) $F_{1,285}$ = 0.8, p=.38<br>sin(FT) $F_{1,285}$ = 4.8, p=.029	R^2 = 0.56<br>AICc = 2740<br>cos(CT) $F_{1,283}$ = 25.4, p<.001<br>sin(CT) $F_{1,283}$ = 318, p<.001<br>cos(FT) $F_{1,283}$ = 4.2, p=.041<br>sin(FT) $F_{1,283}$ = 20.8, p<.001
SMG	R^2 = 0.70<br>AICc = 2900<br>cos(CT) $F_{1,285}$ = 45.2, p<.001<br>sin(CT) $F_{1,285}$ = 621, p<.001	R^2 = 0.04<br>AICc = 3232<br>cos(FT) $F_{1,285}$ = 10.4, p=.001<br>sin(FT) $F_{1,285}$ = 1.9, p=.17	R^2 = 0.74<br>AICc = 2852<br>cos(CT) $F_{1,283}$ = 46.9, p<.001<br>sin(CT) $F_{1,283}$ = 730, p<.001<br>cos(FT) $F_{1,283}$ = 26.0, p<.001<br>sin(FT) $F_{1,283}$ = 21.6, p<.001

Table S2. Total number of fasting intervals (no pellets dropped) of different durations during Ad Lib baseline conditions in both experiments. The number of fasts occurring in the light phase are also shown.

Experiment 1 (8 d baseline in 10 mice = 80 mouse-days)

	Number of fasting intervals					
	Total	>1h	>1.5h	>2h	>3h	>4h
Number	1862	432	192	88	33	13
Percentage	100.0	23.2	10.3	4.7	1.8	0.7
Number in light phase	737	142	134	59	21	12
Percentage	39.6	7.6	7.2	3.2	1.1	0.6

Experiment 2 (10 d baseline in 12 mice = 120 mouse-days)

	Number of fasting intervals					
	Total	>1h	>1.5h	>2h	>3h	>4h
Number	2329	672	371	226	109	53
Percent of total	100.0	28.9	15.9	9.7	4.7	2.3
Number in light phase	951	408	226	132	57	35
Percent of total	40.8	17.5	9.7	5.7	2.4	1.5

Table S3. The number of long (>4h) fasts experienced by each mouse. Note that 20/22 mice had a >4h fast on half or fewer of their baseline days.

		Number of fasts > 4h in light phase									
		0	1	2	3	4	5	6	7	8	9
Expt. 1	# of mice	3	5		1	1					
	Fasts/day	0	0.125		0.375	0.5					
Expt. 2	# of mice	2	3	2	1	2				1	1
	Fasts/day	0	0.1	0.2	0.3	0.4				0.8	0.9
